# Forensic significance of intracardiac heme oxygenase-1 expression in acute myocardial ischemia

**DOI:** 10.1038/s41598-021-01102-y

**Published:** 2021-11-08

**Authors:** Yumi Kuninaka, Yuko Ishida, Mizuho Nosaka, Akiko Ishigami, Akira Taruya, Emi Shimada, Akihiko Kimura, Hiroki Yamamoto, Mitsunori Ozaki, Fukumi Furukawa, Toshikazu Kondo

**Affiliations:** 1 Department of Forensic Medicine, Wakayama Medical University, 811-1 Kimiidera, Wakayama, 641-8509 Japan; 2 Department of Cardiovascular Medicine, Wakayama Medical University, 811-1 Kimiidera, Wakayama, 641-8509 Japan; 3 Department of Neurological Surgery, Wakayama Medical University, 811-1 Kimiidera, Wakayama, 641-8509 Japan; 4 Takatsuki Red Cross Hospital, 1-1-1 Abuno, Takatsuki-shi, Osaka, 569-1096 Japan

**Keywords:** Medical research, Diagnostic markers

## Abstract

Heme oxygenase-1 (HO-1), an inducible stress-response protein, exerts anti-oxidant and anti-apoptotic effects. However, its significance in forensic diagnosis of acute ischemic heart diseases (AIHD) such as myocardial infarction (MI) is still unknown. We examined the immunohistochemical expression of HO-1 in the heart samples to discuss their forensic significance to determine acute cardiac ischemia. The heart samples were obtained from 23 AIHD cases and 33 non-AIHD cases as controls. HO-1 positive signals in cardiomyocyte nuclear were detected in 78.2% of AIHD cases, however, that were detected in only 24.2% control cases with statistical difference between AIHD and non-AIHD groups. In contrast to HO-1 protein expression, there was no significant difference in the appearance of myoglobin pallor regions and leukocyte infiltration in the hearts between AIHD and non-AIHD groups. From the viewpoints of forensic pathology, intracardiac HO-1 expression would be considered a valuable marker to diagnose AIHD as the cause of death.

## Introduction

Ischemic heart disease is the leading cause of death in the worldwide and is the most common cause of sudden cardiac death (SCD)^1,2^. In the cases of SCD, the postmortem diagnosis of acute myocardial ischemia is an important issue for both clinical physicians and forensic pathologists if death occurs within a short period of time after the onset of ischemic heart attack.

Myocardial infarction (MI), pathologically defined as myocardial cell death due to prolonged ischemia, is most representative acute ischemic heart disease (AIHD). After the onset of myocardial ischemia, histological cell death does not occur immediately, and it takes a finite period of time to develop—as little as 20 min, or less in some animal models^3–5^. Depending on the sensitivity of cardiomyocytes, macroscopic or microscopic postmortem examination takes several hours to identify myocardial necrosis. Thus, the evaluation of early myocardial damage using routine histological examination such as HE staining is only possible in death cases at least several hours passed after the onset of ischemic damage.

To identify early signs of cardiac ischemia, histochemical techniques have been proposed^6–12^. However, these methods are not suitable for routine use, and their specificity and sensitivity are controversial because positive results may be sometimes associated with agonal factors and postmortem changes^13,14^. Recent works have immunohistochemically investigated, some markers such as fibronectin, C5b-9 and myoglobin which accumulate in or leak from cardiomyocytes after ischemia. However, these molecules almost failed to be detected in the very early phase of myocardial ischemia^5,15–18^.

Heme oxygenase-1 (HO-1, HSP32) is a vital heme degradative enzyme and inducible stress protein^19–21^. Bilirubin and carbon monoxide (CO) that are reaction products of HO-1 are protective against ischemia-induced injury such as myocardial infarction, ischemia–reperfusion injury, and post-infarct structural remodeling^22–24^. Oxidative stress associated with over-vasoconstriction is the major pathogenic mechanism in the various heart pathologies. Nuclear translocation of HO-1 induced by oxidative stress resulted in the promoted interaction of HO-1 with the redox-sensitive transcription factor Nrf2^25^. The observations described previously suggest that HO-1 had a role in the pathogenesis of AIHD. However, the knowledge of their involvement in AIHD and their significance in forensic diagnose are insufficient. Therefore, in the present study, we examined intracardiac HO-1 protein expression in forensic autopsy samples and discussed its availability as a potential indicator for postmortem diagnosis of AIHD.

## Results

### Histopathological and immunohistochemical analyses in autopsy samples

**Figure 1:**
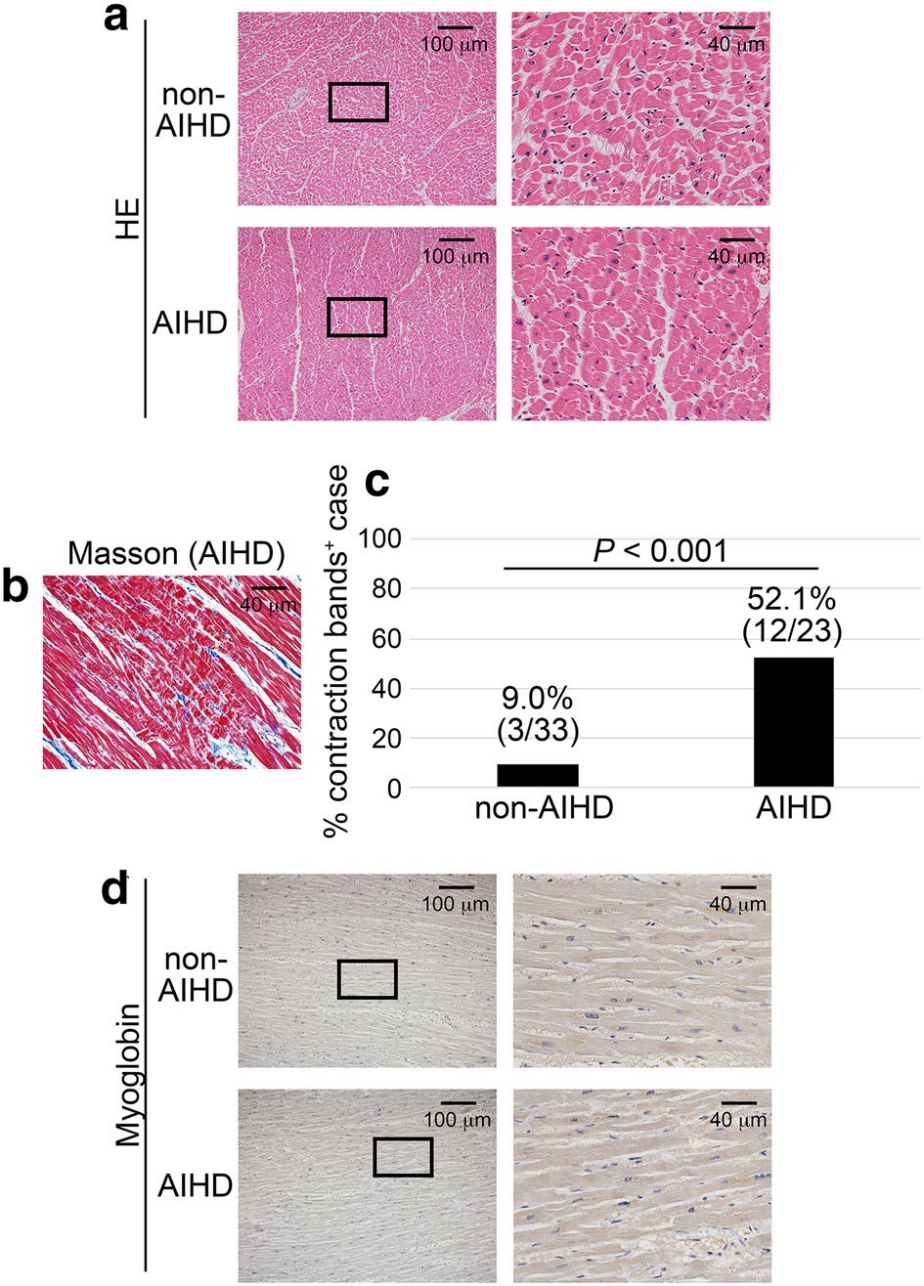
Histopathological and immunohistochemical analysis of the human hearts. (**a**) HE staining. Representative results from non-AIHD (Drowning) and AIHD groups were shown here. (**b**) Contraction bands on the heart of AIHD group (Masson trichrome staining). (**c**) Percentage of cases where contraction bands were present. (**d**) Immunohistochemical analysis by using anti-myoglobin. Representative results from non-AIHD (Drowning) and AIHD groups were shown here.

Histopathologically, HE staining showed that there was no specific histological sign of MI in both AIHD and non-AIHD groups (Fig. 1a and Supplemental Fig. 1). However, contraction bands could be confirmed by Masson trichrome staining in some of AIHD groups (Fig. 1b), and there was a significant difference of percentage of the case where contraction band existed evidently between AIHD and non-AIHD groups (52.1% vs. 9.0%, Fig. 1c). Moreover, the immunohistochemical staining of myoglobin revealed no differences in the appearance of myoglobin pallor regions between AIHD and non-AIHD groups (Fig. 1d and Supplemental Fig. 2).

**Figure 2:**
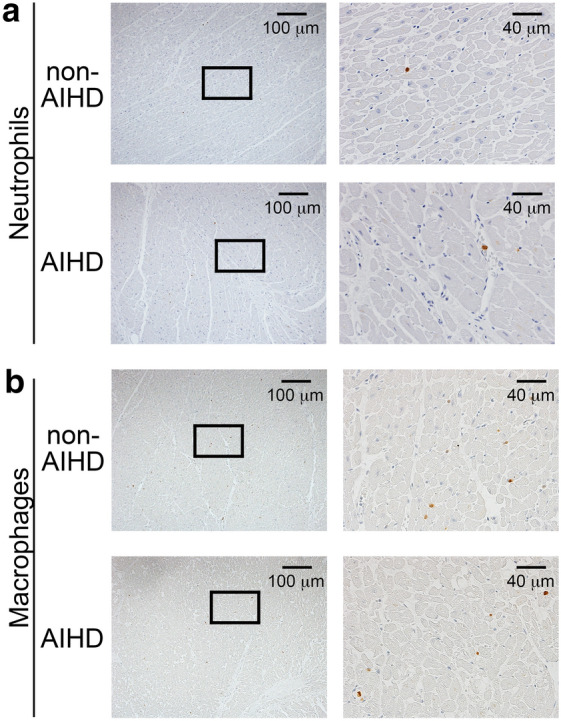
Detection of leukocyte infiltration in the non-AIHD and AIHD hearts. (**a**,**b**) Immunohistochemical analysis by using anti-MPO for neutrophils (**a**) and anti-Macrophage Marker for macrophages (**b**). Representative results from non-AIHD (Drowning) and AIHD groups were shown here.

### Intracardiac leukocyte infiltration

Intracardiac leukocyte recruitment is also one of the hallmarks in AIHD. Thus, we examined the degrees of intracardiac leukocyte recruitment. Subsequently, immunohistochemical analyses demonstrated that few MPO^+^ neutrophils (Fig. 2a and Supplemental Fig. 3) and macrophages (Fig. 2b and Supplemental Fig. 4) were present in the control hearts. In AIHD hearts, neutrophils and macrophages were observed to a similar extent as non-AIHD (Fig. 2). These observations implied that the evaluation of leukocyte recruitment would not be powerful clue for the diagnosis of early phase of AIHD.

**Figure 3:**
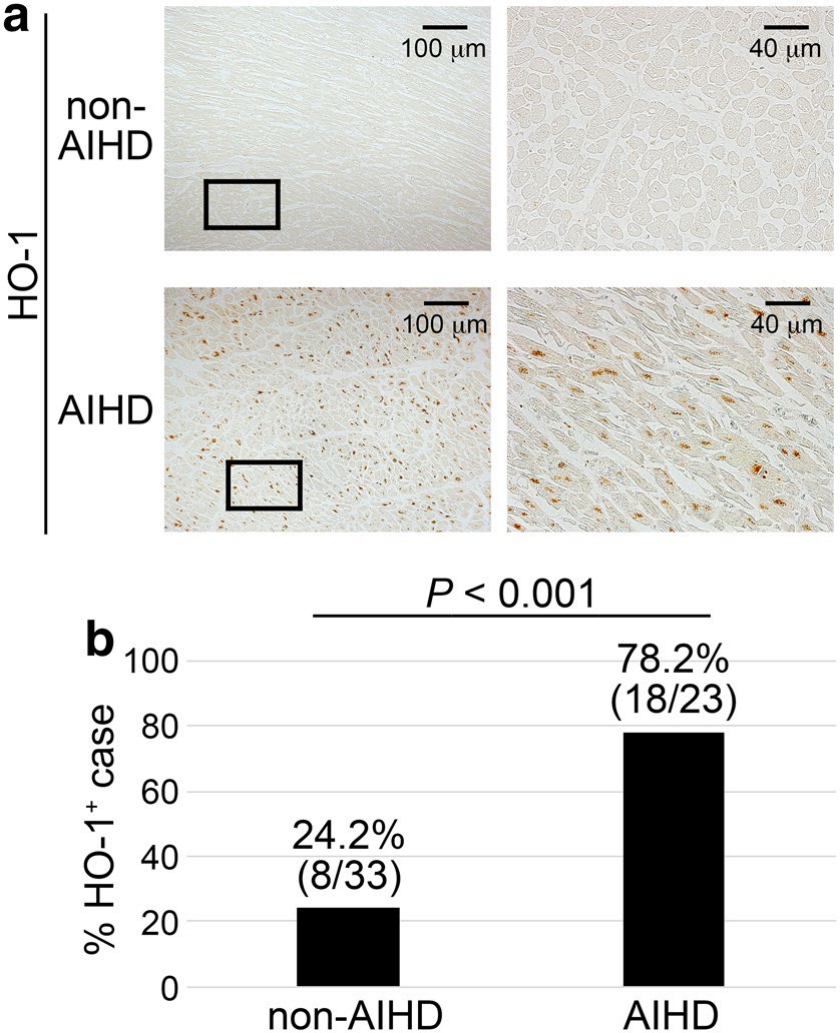
The expression of HO-1 on the human hearts. (**a**) Immunohistochemical analysis by using anti-HO-1. Representative results from the hearts of non-AIHD (Drowning) and AIHD groups were shown here. (**b**) Percentage of cases where HO-1-positive nuclei of cardiomyocytes were exist.

**Figure 4:**
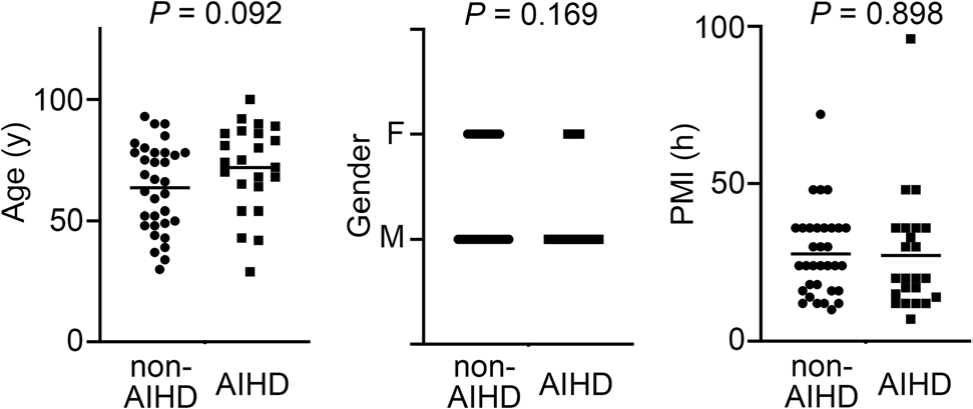
The relation between age, gender, or PMI, and non-AIHD and AIHD groups. These results were obtained with Spearman’s correction coefficient by rank test.

### Enhanced intracardiac HO-1 expression in AIHD group

We could observe intracardiac HO-1 expression in both groups. The immunohistochemical expression of HO-1 was more intense and diffuses in the nuclei of cardiomyocytes in AIHD group, compared with non-AIHD group ones (Fig. 3a and Supplemental Fig. 5). The comparison between AIHD and non-AIHD groups showed significant difference of the number of HO-1 expressed case (Fig. 3b). There were no significant differences on age, gender, or PMI for HO-1 protein expressions (Fig. 4).

## Discussion

The post-mortem diagnosis of SCD can be difficult if it is based only on routine investigation as heart macroscopic findings and HE analyses. As a method for detecting myocardial ischemia, visualization of molecular change by immunohistochemical examination has been proposed, but its application depends on the availability of a specific and sensitive marker^15,26,27^. Until now, several lines of accumulating evidence implied the possibility usefulness of C5b-9 and fibronectin in forensic diagnosis of myocardial ischemia through both human samples and animal models^15,27–31^. However, no single immunohistochemical reaction is ideal for diagnosis early myocardial ischemia. The present study demonstrated the usefulness of HO-1 for the postmortem diagnosis of SCD.

HO-1 is a rapidly inducible protein which degrades heme to biliverdin, ferrous iron, and CO in order to protect cells from oxidative stress^19–21^. HO-1 mitigate cellular injury by its anti-oxidant, -apoptotic, and -inflammatory effects^21,32–34^. HO-1-mediated cytoprotection reflects the effects of its catalytic products especially CO because CO can rescue the injurious effects of HO-1 deficiency^21,32–35^. Although short-term HO-1-mediated cytoprotective effects in heart of animals have been reported^36,37^, it was unknown whether HO-1 could be an useful indicator of postmortem diagnosis of human AIHD. Novo et al.^38^ determined serum levels of HO-1 in acute MI patients to assess their clinical significance and potential prognostic value. They found that serum HO-1 level was increased in patients of coronary artery diseases, which is in accordance with the cardioprotective role of HO-1^39^. In the present study, we found that the intranuclear HO-1 expression in cardiomyocytes were detected in 78.2% (18 cases/23 cases) of AIHD group suggesting that HO-1 expression can be a marker of early ischemia.

In individuals with an ischemic injury for several hours, the myocardial alterations can be detected as follows: necrotic clotting, wavy fibers, hypereosinophilic anucleated myocytes, and early inflammatory infiltration^40^. The contraction bands also have been considered as a sign of ischemia and a histological hallmark of adrenergic stress and/or reperfusion injury^41–43^. In fact, although the frequency was low, about half of AIHD group (12 cases/23 cases, 52.1%) had contraction bands with a significant difference, compared with non-AIHD one (3 cases/33 cases, 9.0%).

Activation of inflammation is important to clear the damaged myocardium. Diffuse leukocyte infiltration is first observed after 9 h from the onset of myocardial ischemia^44,45^. Our data showed few neutrophils and macrophages in the both AIHD and non-AIHD hearts with no significant difference, suggesting that detection of leukocytes in the hearts would not be suited for postmortem diagnosis of early phase of AIHD.

The early loss of myoglobin within 6 h from the onset of the symptoms is clearly documented in immunohistochemical studies on human samples^26,27,46,47^. This marker has been tested enough sensitive and specific for the detection of AIHD lasting for several hours, however, may not be suitable for detection of very early myocardial ischemic damage.

C5b-9 is most commonly used to support the final diagnosis of SCD because it can reveal areas of myocardial necrosis^5,15,27,30^. This property of C5b-9 is due to its direct involvement in the complement cascade. Indeed, it has been reported that the C5b-9 complex, which is known to form under ischemic conditions, may directly contribute to cardiomyocyte damage^48^. In addition, immunohistochemical demonstrations of fibronectin have been shown to be useful in the localization and diagnosis of early ischemic myocardial necrosis^5,15,27,31^. Fibronectin leaks from damaged capillaries and extracellular matrix to damaged myocytes during ischemia. Hu et al. reported experimentally observing a positive reaction of fibronectin in the cytoplasm of necrotic myocytes 30–60 min after coronary artery occlusion^49^.

For the immunohistochemical evaluation of C5b-9 and fibronectin in the previous studies, positive staining was graded^5,15,27,31^. On the other hand, the interpretation of HO-1 staining in our study is evaluated as positive or negative, which imply that results may be less influenced by each investigator, resulting in less variation of the interpretation. This suggests that our study would be more simple and advantageous in forensic practices, compared with the previous studies.

In this study, we proposed a useful marker HO-1 in the forensic diagnosis of acute myocardial ischemia. In forensic practices, it is difficult to make definite evaluation of the cause of death, wound ages or wound vitality using a single marker. In line with this, no single immunohistochemical reaction is ideal for diagnosis of early myocardial ischemia in the aspects of forensic safety. Taken together, the combined analysis using several different markers such as HO-1 as well as C5b-9 and fibronectin could provide more objective and accurate information for the evaluation of early myocardial damage, eventually contributing to the advance of postmortem diagnosis of AIHD when macroscopic or microscopic evidence is insufficient.

## Methods

### Antibodies (Abs)

The following polyclonal or monoclonal Abs (pAbs or mAbs) were used for immunohistochemical analyses in the present study: goat anti-human HO-1 pAbs (1:200, #ADI-SPA-896, Enzo Life Science, Farmingdale, NY), rabbit anti-myeloperoxidase (MPO) pAbs (1:100, RB-373-A, Lab Vision/Neo Markers, Fremont, CA), mouse anti-human macrophage marker (CD68) mAb (1:100, clone MAC387, sc-66204, Santa Cruz, Dallas, TX), rabbit anti-human myoglobin pAbs (1:2000, #A0324, DAKO, Santa Clara, CA).

### Human myocardial tissues

A total of 56 human forensic autopsy cases (38 males and 18 females) with a postmortem interval (PMI) of less than 96 h were selected based on autopsy documents. The individual ages ranged from 29 to 100 years (mean age, 67.6 years). In each case, the cause of death was carefully diagnosed on the basis of complete autopsy, histology, toxicology, and diatom test. Cases were divided into two groups as follows: 23 acute ischemic heart disease with advanced sclerosis and/or stenosis in coronary artery and 33 others including drowning (18 cases), asphyxia (3 case), fire death (3 cases), hanging (2 cases), acute drug poisoning (3 cases), and one each of acute carbon monoxide poisoning, traumatic shock, sepsis, severe head injury. The detail profiles of all cases (gender, age, PMI) are shown in Table 1.

**Table 1:** Forensic autopsy cases.

Cause of death	No	Male/female	Age (years)		PMI (h)	
			Range	Mean	Range	Mean
AIHD	23	18/5	29–100	71.8	7–96	27.2
Drowning	18	11/7	39–93	73	10–48	26
Asphyxia	3	1/2	37–54	47.6	12–36	20.6
Fire death	3	2/1	62–90	76.6	12–24	17.3
Hanging	2	2/0	49–52	50.5	36–48	42
Acute drug poisoning	3	2/1	30–44	36	24–48	36
Acute carbon poisoning	1	1/0	48	48	30	30
Traumatic shock	1	0/1	43	43	12	12
Sepsis	1	0/1	50	50	30	30
Severe head injury	1	1/0	59	59	72	72
Total	56	38/18	29–100	67.6	7–96	27.5

### Histopathological analyses

Heart samples were fixed in 4% formaldehyde buffered with PBS and then embedded with paraffin. Sections were stained with hematoxylin–eosin (HE) and Masson trichrome for histological analysis. Immunohistochemical analysis was also performed using anti-HO-1, -MPO, -Macrophage Marker, or -myoglobin Abs. Deparaffinized sections were immersed in 0.3% H_2_O_2_ in methanol for 30 min to eliminate endogenous peroxidase activities. The sections were further incubated with PBS containing 1% normal serum corresponding to the secondary Abs and 1% BSA to reduce nonspecific reactions. The sections were incubated with primary Abs at 4 °C overnight. Normal rabbit, goat, or mouse IgG was used as negative control. After incubation with biotinylated secondary Abs, immune complexes were visualized using the Catalyzed Signal Amplification System (Dako Cytomation, Kyoto, Japan) according to the manufacturer’s instructions.

### Morphometrical analysis

Morphometrical analysis was performed with immunohistochemical findings. Cases in which HO-1-positive cells were uniformly observed in myocardial samples were defined as HO-1^+^ cases. These analyses were performed by two investigators with no prior knowledge of the samples.

### Statistical analysis

Comparison of two groups was performed using two-side unpaired Student’s *t* test to identify significant difference. *P* < 0.05 was considered as significant. All statistical analyses were performed using Statcel3 software under the supervision of a medical statistician.

## Supplementary Information

Supplementary Information.

## Data Availability

The authors declare that all data are available in the article file, or available from the authors upon reasonable request.
