# Supplementary material for: Forensic significance of intracardiac heme oxygenase-1 expression in acute myocardial ischemia
Source: Sci Rep. 2021 Nov 8;11:21828. doi: 10.1038/s41598-021-01102-y (PMC8575909; doi:10.1038/s41598-021-01102-y)
Supplement: Supplementary file 1 — Supplementary Information. [file 41598_2021_1102_MOESM1_ESM.pdf]

Forensic significance of intracardiac heme oxygenase-1 expression in acute myocardial ischemia

Yumi Kuninaka[1,*], Yuko Ishida[1,*,**], Mizuho Nosaka[1], Akiko Ishigami[1], Akira Taruya[2], Emi Shimada[1], Akihiko Kimura[1], Hiroki Yamamoto[1], Mitsunori Ozaki[3], Fukumi Furukawa[1,4], Toshikazu Kondo[1,**]

[1]Department of Forensic Medicine, Wakayama Medical University, 811-1 Kimiidera, 641-8509 Wakayama, Japan

[2]Department of Cardiovascular Medicine, Wakayama Medical University, 811-1 Kimiidera, 641-8509 Wakayama, Japan

[3]Department of Neurological Surgery, Wakayama Medical University, 811-1 Kimiidera, 641-8509 Wakayama, Japan

[4]Takatsuki Red Cross Hospital, 1-1-1 Abuno, Takatsuki-shi, Osaka 569-1096, Japan

Running title: Heme oxygenase-1 in acute myocardial ischemia

*Both authors equally contributed to this work.

**Corresponding authors: Yuko Ishida and Toshikazu Kondo

E-mail: kondot@wakayama-med.ac.jp

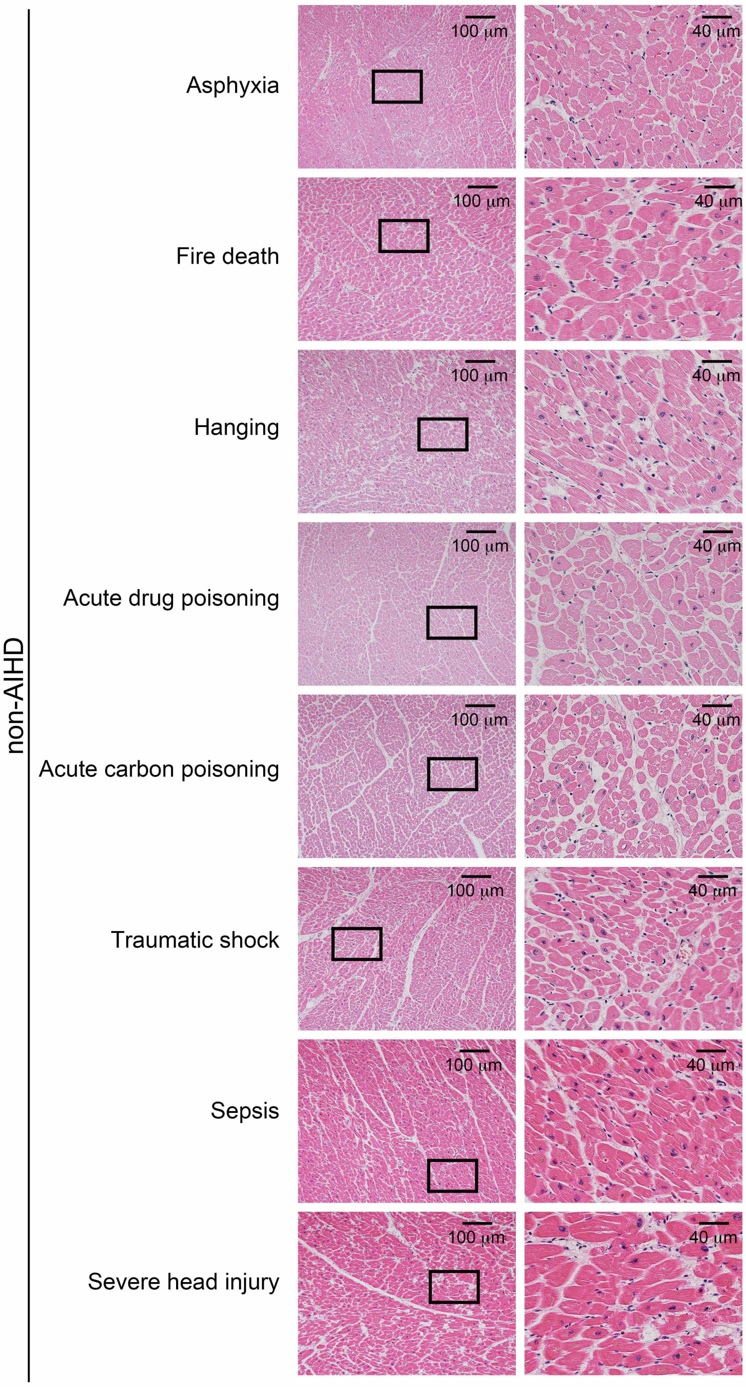

Supplemental figure 1. Histopathological analysis of the human hearts (HE staining). Representative results from non-AIHD (asphyxia, fire death, hanging, acute drug poisoning, acute carbon poisoning, traumatic shock, sepsis, and severe head injury) were shown here.

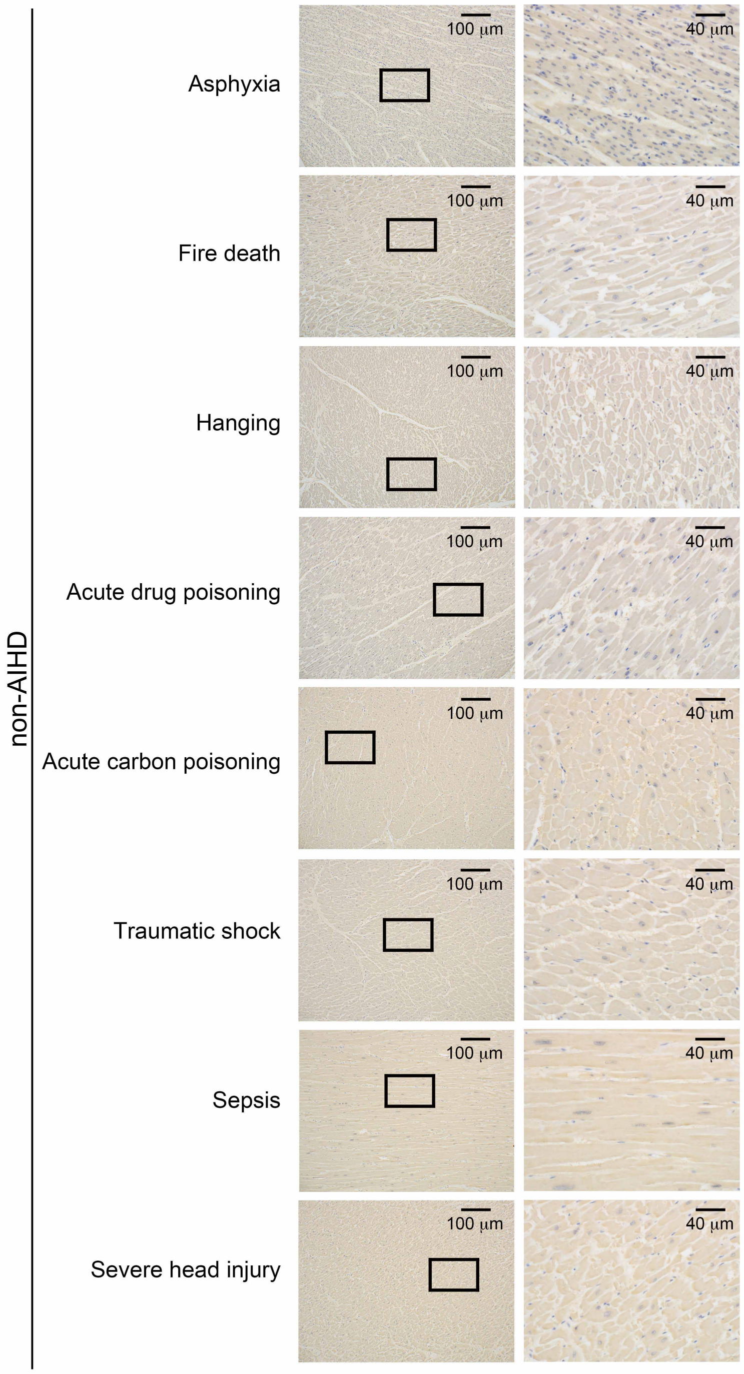

Supplemental figure 2. Immunohistochemical analysis by using anti-myoglobin. Representative results from non-AIHD (asphyxia, fire death, hanging, acute drug poisoning, acute carbon poisoning, traumatic shock, sepsis, and severe head injury) were shown here.

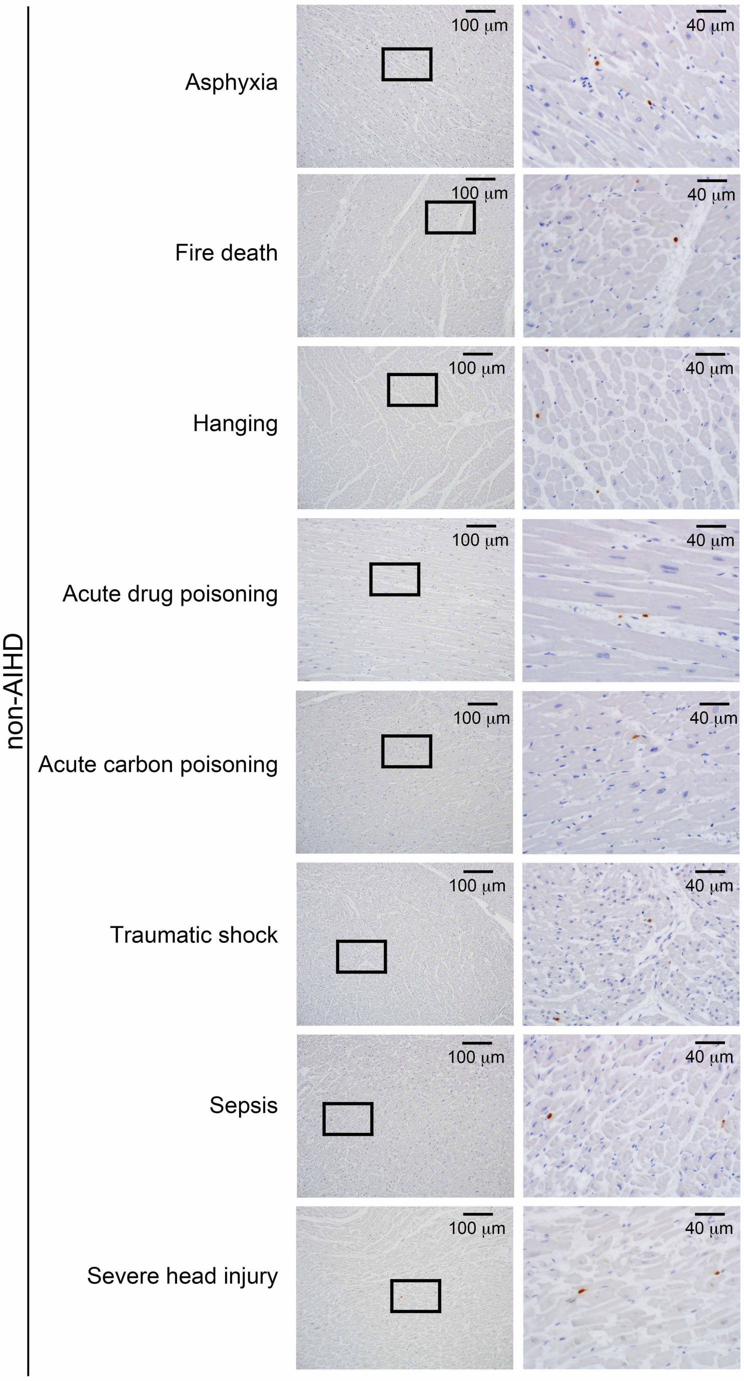

Supplemental figure 3. Detection of neutrophil infiltration in the non-AIHD hearts. Immunohistochemical analysis by using anti-MPO for neutrophils. Representative results from non-AIHD (asphyxia, fire death, hanging, acute drug poisoning, acute carbon poisoning, traumatic shock, sepsis, and severe head injury) were shown here.

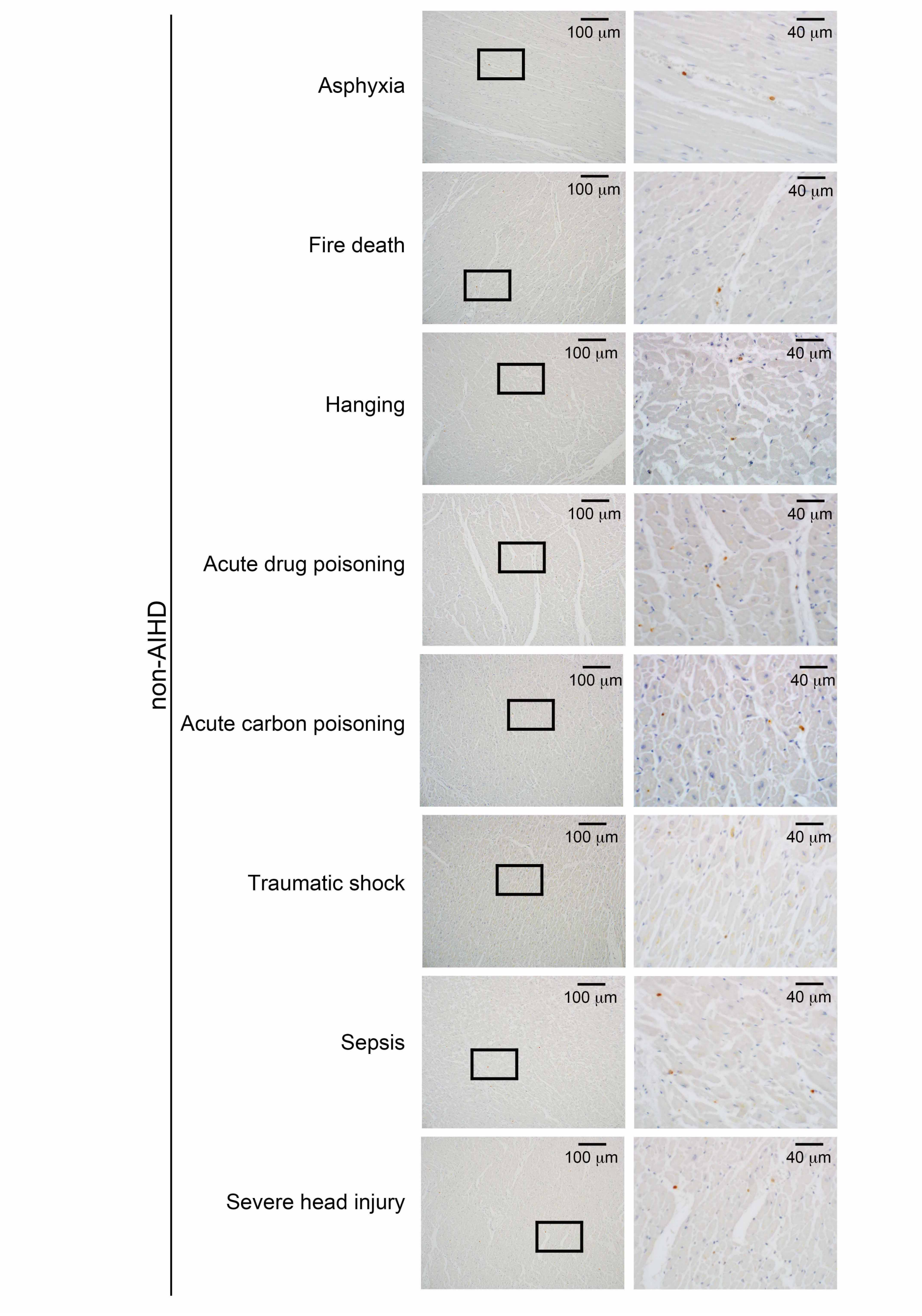

Supplemental figure 4. Detection of macrophage infiltration in the non-AIHD hearts. Immunohistochemical analysis by using anti- Macrophage Marker for macrophages. Representative results from non-AIHD (asphyxia, fire death, hanging, acute drug poisoning, acute carbon poisoning, traumatic shock, sepsis, and severe head injury) were shown here.

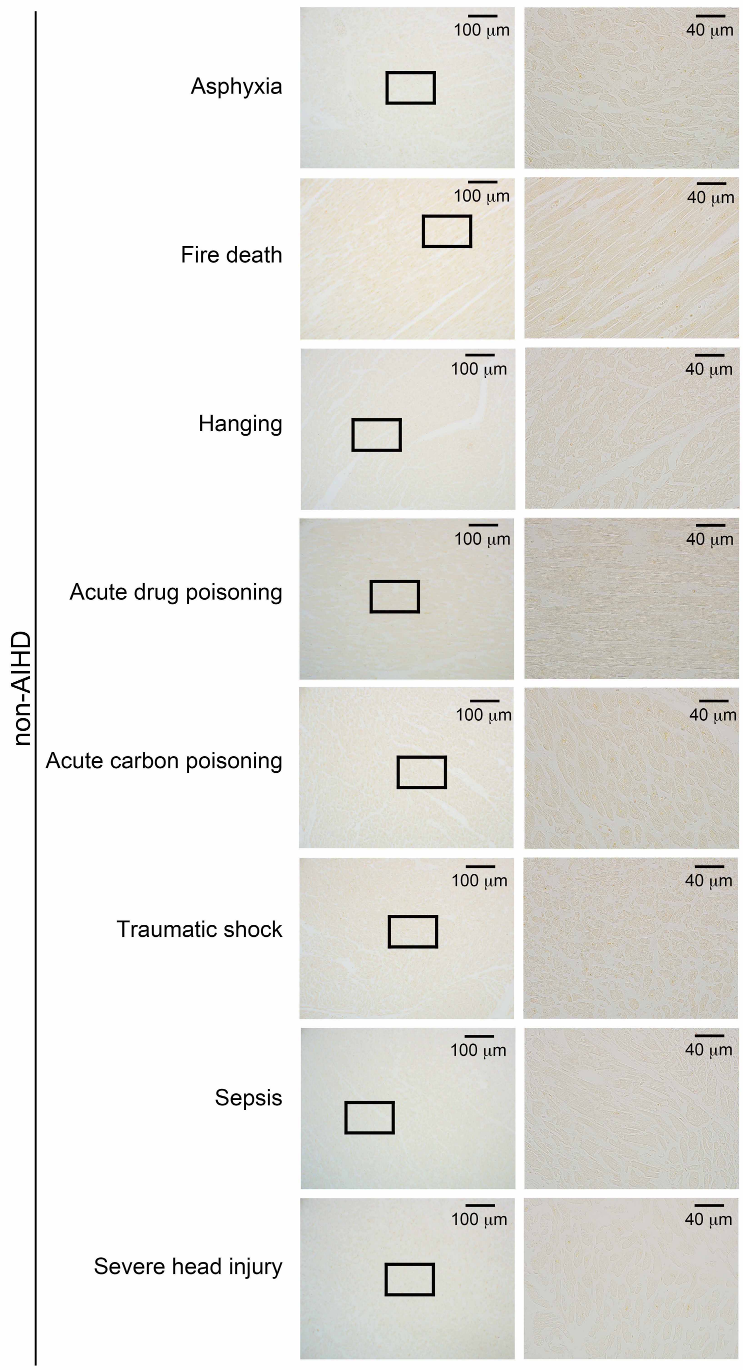

Supplemental figure 5. Immunohistochemical analysis by using anti-HO-1. Representative results from the hearts of non-AIHD (asphyxia, fire death, hanging, acute drug poisoning, acute carbon poisoning, traumatic shock, sepsis, and severe head injury) were shown here.
